# Predicting non-small cell lung cancer prognosis by fully automated microscopic pathology image features

**DOI:** 10.1038/ncomms12474

**Published:** 2016-08-16

**Authors:** Kun-Hsing Yu, Ce Zhang, Gerald J. Berry, Russ B. Altman, Christopher Ré, Daniel L. Rubin, Michael Snyder

**Affiliations:** 1 Biomedical Informatics Program, Stanford University, 1265 Welch Road, MSOB, X-215, MC 5479, Stanford 94305-5479, California, USA; 2 Department of Genetics, Stanford University, 300 Pasteur Dr, M-344, Stanford 94305-5120, California, USA; 3 Department of Computer Science, Stanford University, 353 Serra Mall, Stanford 94305-9025, California, USA; 4 Department of Pathology, Stanford University, 300 Pasteur Dr, L235, Stanford 94305, California, USA

## Abstract

Lung cancer is the most prevalent cancer worldwide, and histopathological assessment is indispensable for its diagnosis. However, human evaluation of pathology slides cannot accurately predict patients' prognoses. In this study, we obtain 2,186 haematoxylin and eosin stained histopathology whole-slide images of lung adenocarcinoma and squamous cell carcinoma patients from The Cancer Genome Atlas (TCGA), and 294 additional images from Stanford Tissue Microarray (TMA) Database. We extract 9,879 quantitative image features and use regularized machine-learning methods to select the top features and to distinguish shorter-term survivors from longer-term survivors with stage I adenocarcinoma (*P*<0.003) or squamous cell carcinoma (*P*=0.023) in the TCGA data set. We validate the survival prediction framework with the TMA cohort (*P*<0.036 for both tumour types). Our results suggest that automatically derived image features can predict the prognosis of lung cancer patients and thereby contribute to precision oncology. Our methods are extensible to histopathology images of other organs.

## Abstract (web summary)

Diagnosis of lung cancer through manual histopathology evaluation is insufficient to predict patient survival. Here, the authors use computerized image processing to identify diagnostically relevant image features and use these features to distinguish lung cancer patients with different prognoses.

## Graphical abstract

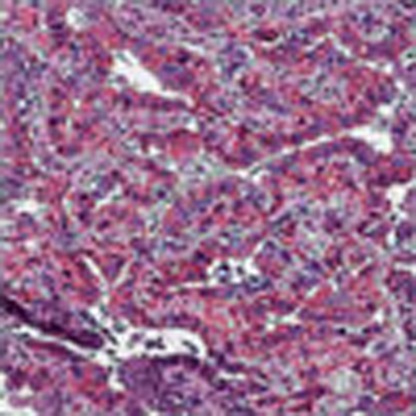

Lung cancer is the most prevalent cancer and the leading cause of cancer-related deaths worldwide, resulting in more than 1.4 million deaths annually^1^,^2^. Evaluation of the microscopic histopathology slides by experienced pathologists is indispensable to establishing the diagnosis^3^,^4^,^5^ and defines the types and subtypes of lung cancers, including the two major types of non-small cell lung cancer: adenocarcinoma and squamous cell carcinoma^6^,^7^,^8^. The distinction of squamous cell carcinoma from adenocarcinoma is important for chemotherapeutic selection, because certain antineoplastic agents are contraindicated for squamous cell carcinoma patients because of decreased efficacy^9^ or increased toxicity^10^. In addition, more adenocarcinoma patients possess genetic aberrations with available targeted therapy, such as EGFR mutations and ALK rearrangements^11^,^12^,^13^. Certain histological features, such as pathology grade, have been associated with survival outcomes in some studies^14^,^15^. Prompt and meticulous inspection of tumour histomorphology is critical to patient care, and determination of relevant prognostic markers is the key to personalized cancer management. For example, patients with poorer prognoses may benefit from closer follow-up, more aggressive form of treatment, and advance care planning^16^,^17^.

Currently, lung cancer samples are manually evaluated for their histological features by light microscopy. However, qualitative evaluation of well-established histopathology patterns alone (such as the classification of tumour grades) is insufficient for predicting the survival outcomes of patients with lung adenocarcinoma or lung squamous cell carcinoma^18^,^19^, and even the best-characterized histopathology features only achieve modest agreements among experienced pathologists. As an illustration, the inter-observer agreement for features that define the types of non-small cell lung cancer is moderate (*κ*=0.48–0.64)^20^, and the diagnostic agreement for classifying adenocarcinomas and squamous carcinomas is also relatively low (*κ*=0.41–0.46 among community pathologists, *κ*=0.64–0.69 among pulmonary pathology experts and *κ*=0.55–0.59 among all pathologists under study)^21^. Poorer tumour differentiation and poorer slide quality were associated with lower diagnostic agreement^21^. Several recent studies have attempted to define additional visual features for prognostic prediction for patients with lung adenocarcinoma^4^,^22^,^23^ or lung squamous cell carcinoma^24^,^25^. However, there is still considerable room for improvement for the inter-rater agreements of these features^26^,^27^,^28^. Subjective or erroneous evaluation of histopathology images may lead to poor therapeutic choice, which results in decreased survival and loss of quality of life in numerous patients^29^.

Computerized image processing technology has been shown to improve efficiency, accuracy and consistency in histopathology evaluations, and can provide decision support to ensure diagnostic consistency^30^. Automated histopathological analysis systems also have been proven to be valuable in prognostic determinations of various malignancies, including breast cancer^31^, neuroblastoma^32^, lymphoma^33^ and pre-cancerous lesions in the esophagus^34^. Automated systems can identify candidate regions that require further diagnostic assessment and propose novel image features useful for prognosis. Current clinical practice could thus benefit greatly from the development and incorporation of such systems into clinical care^31^,^32^. With the recent availability of digital whole-slide images^30^, there is now an opportunity for systematic analysis of the microscopic morphology of lung cancer cells, whose structural diversity had previously posed a great challenge for automated analysis^35^,^36^. In particular, there is the possibility of identifying previously unrecognized image features that correlate with patients' prognoses, and potentially guide treatment decisions^31^.

In this study, we aim to improve the prognostic prediction of lung adenocarcinoma and squamous cell carcinoma patients through objective features distilled from histopathology images. We design a fully automated informatics pipeline to extract objective quantitative image features, assess the diagnostic utility of the feature sets, build classifiers to distinguish lung cancers with different survival outcomes, discover novel image features that predicted patient prognosis and validate the results in an independent data set. Our methods may ultimately provide prognostic information for the patients, and contribute to precision medicine of lung cancer.

## Results

### Patient characteristics and fully automated image features

We obtained 2,186 haematoxylin and eosin (H&E) stained whole-slide histopathology images from The Cancer Genome Atlas (TCGA)^37^,^38^, encompassing lung adenocarcinoma and lung squamous cell carcinoma as well as adjacent benign tissue. All images captured at × 40 magnification were tiled with open microscopy environment tools^39^. To target regions with pathological changes, our automated pipeline skipped regions with relatively sparse cellularity such as alveolar spaces and selected the 10 densest tiles per image for further analysis. We also acquired 294 tissue microarray images from the Stanford Tissue Microarray (TMA) Database^40^, with one representative histopathology image selected by pathologists for each of the 227 lung adenocarcinoma and 67 lung squamous cell carcinoma patients. Patient characteristics of both the TCGA and TMA cohorts are summarized in Tables 1 and 2, respectively.

To extract objective morphological information from thousands of images, we built a fully automated image-segmentation pipeline to identify the tumour nuclei and tumour cytoplasm from the histopathology images using the Otsu method^41^ (see Methods for details), and extracted quantitative features from the identified tumour nuclei and cytoplasm (Supplementary Fig. 1). Our fully automated pipeline reliably identified most tumour cells and tumour nuclei, and the results were consistent across different slides and images from different batches (Supplementary Fig. 2). A total of 9,879 quantitative features were extracted from each image tile with CellProfiler^42^,^43^. Types of image features included cell size, shape, distribution of pixel intensity in the cells and nuclei, as well as texture of the cells and nuclei. Supplementary Table 1 provides a list of feature categories included in this study.

### Image features accurately identify tumour parts

**Figure 1:**
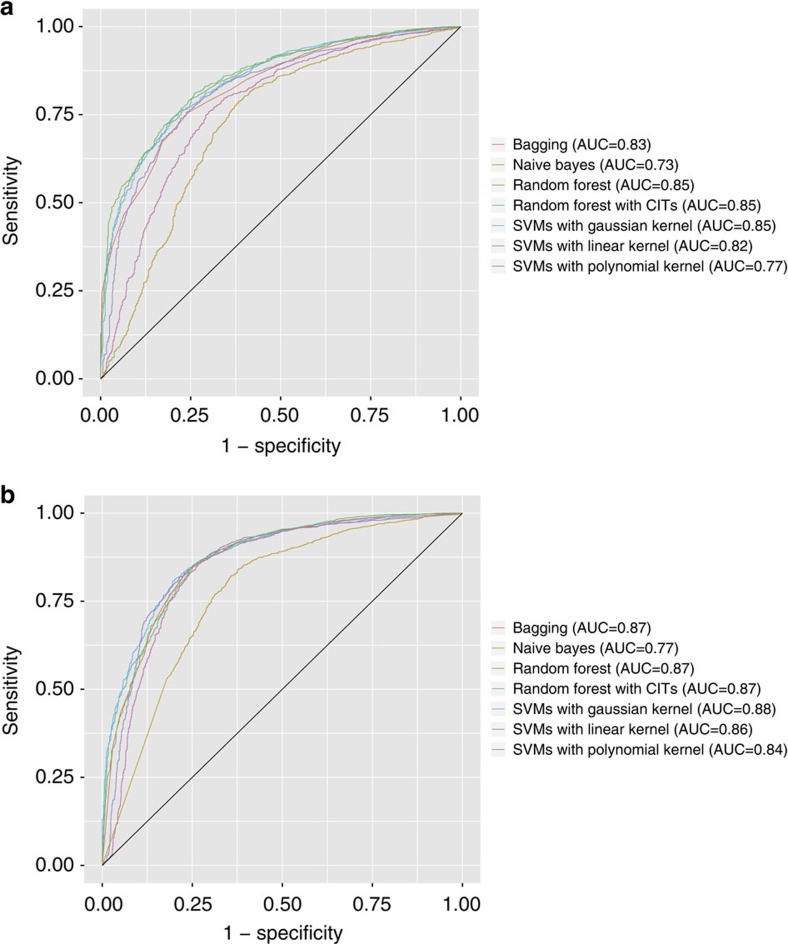
Quantitative image features accurately distinguished malignancies from adjacent dense normal tissues. (**a**) ROC curves for classifying lung adenocarcinoma versus adjacent dense normal tissues in the TCGA test set. Classifiers with 80 features attained average AUC of 0.81. (**b**) ROC curves for classifying lung squamous cell carcinoma from adjacent dense normal tissues in the TCGA test set. Classifiers with 80 features attained average AUC of 0.85. The performance of different classifiers is shown. CIT, conditional inference trees; ROC, receiver operator characteristics.

To determine if the quantitative image features were biologically relevant, we first examined if they could distinguish malignancy from normal adjacent tissue (inflammation, atelectasis or lymphocytic infiltration in the absence of tumour cells) for the TCGA cohort. We used seven classifiers: naive Bayes, support vector machines (SVM) with Gaussian kernel, SVM with linear kernel, SVM with polynomial kernel, bagging for classification trees, random forest utilizing conditional inference trees^44^ and Breiman's random forest^45^. The TCGA data set was randomly partitioned into distinct training and test set, with models built and optimized through the training data and classification performance evaluated through the test set. This process was repeated 20 times to ensure the robustness of our classifiers. Our classifiers achieved an average area under the receiver operating characteristic curve (AUC) of 0.81 (best classifiers: SVM with Gaussian kernel, random forest utilizing conditional inference trees, and Breiman's random forest (AUC=0.85). The performance of these three classifiers did not differ significantly (analysis of variance (ANOVA) test *P* value=0.8514)) in distinguishing between adenocarcinoma and adjacent dense benign tissue when using the top 80 quantitative features (Fig. 1a and Supplementary Table 2). When classifying squamous cell carcinoma with adjacent benign tissue, the AUCs of our classifiers with 80 features were >0.85 (Fig. 1b and Supplementary Table 2). The performance of the top three classifiers did not differ much (ANOVA test *P* value=0.31). In general, the top quantitative features were Haralick features of the nuclei (sum variance, difference variance, correlation coefficient of adjacent pixels), radial distribution of pixel intensity and intensity mass displacement of the cytoplasm.

### Image features distinguish tumour types in both cohorts

**Figure 2:**
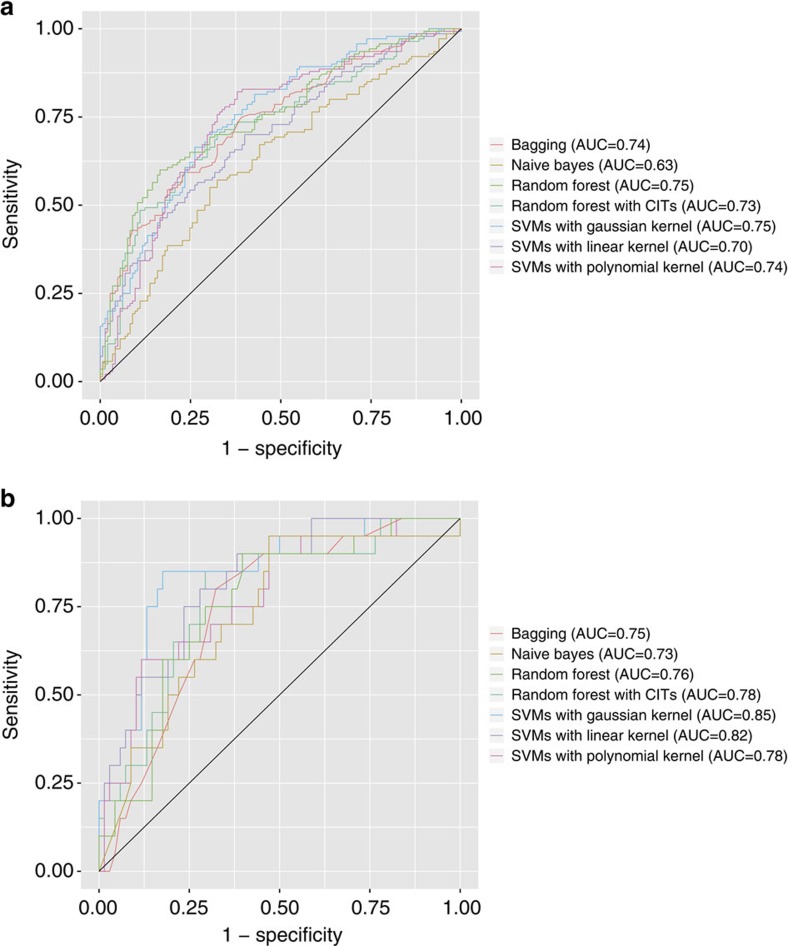
Quantitative image features successfully distinguished histopathology images of lung adenocarcinoma from those of lung squamous cell carcinoma. (**a**) ROC curves for classifying the two malignancies in the TCGA test set. Most classifiers achieved AUC>0.7. (**b**) ROC curves for classifying the two malignancies in the TMA test set. Most classifiers achieved AUC >0.75, indicating that our informatics pipeline was successfully validated in the independent TMA data set. The performance of different classifiers is shown. CIT, conditional inference trees; ROC, receiver operator characteristics.

To further validate the biological relevance of the quantitative features, we applied our classifiers to distinguish between adenocarcinoma and squamous cell carcinoma using the same set of fully automated features in both TCGA and TMA data sets. Our results showed that using 240 features selected by their utility in this task (assessed through the information gain ratio measurement), our best classifiers, including SVMs with Gaussian kernel and random forest classifiers, attained an AUC of above 0.75 in the TCGA data set (average of all classifiers: 0.72; Fig. 2a and Supplementary Table 3). The performance of the top classifiers did not differ significantly (ANOVA test *P* value=0.08). The top quantitative features selected by information gain ratio included Haralick texture features of the nuclei (sum entropy, InfoMeas1, difference variance, angular second moment), edge intensity of the nuclei, texture features of the cytoplasm and intensity distribution of the cytoplasm. Some of the feature groups overlapped with those that were used to distinguish between benign and malignant lesions. For instance, Haralick texture features such as sum entropy and difference variance were among the top features in both classification tasks.

The relevance of our quantitative image features for diagnostic classification was also validated in the TMA data set. Utilizing the same informatics pipeline on these samples, most of the classifiers achieved AUC around 0.78 (SVM with Gaussian kernel has the highest AUC of 0.85; the performance of the top three classifiers did not differ significantly (ANOVA test *P* value=0.13)), indicating the robustness of our informatics method (Fig. 2b and Supplementary Table 3). The slightly higher AUC in the TMA samples relative to the TCGA samples may be due to the manual selection of representative views by the pathologist, whereas the entire slide was used for the TCGA samples. The top quantitative features included texture features in the tumour nucleus and cytoplasm, and radial distribution of pixel intensity.

### Image features predict stage I adenocarcinoma survival

**Figure 3:**
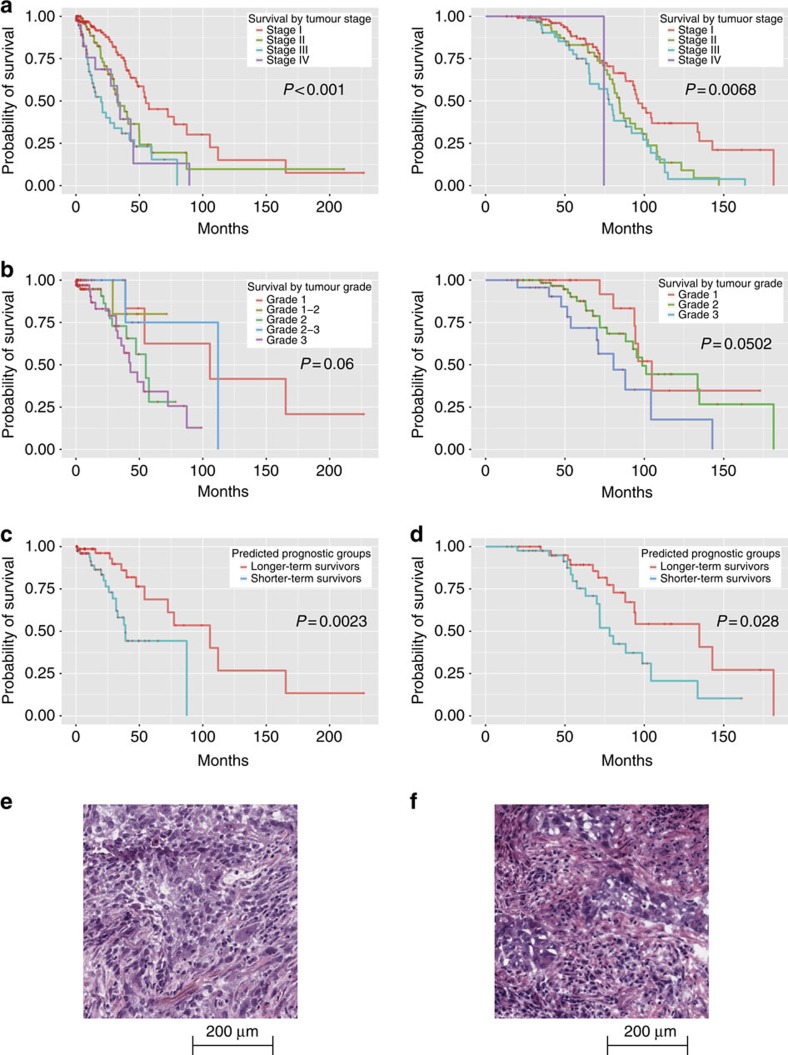
Quantitative image features predicted the survival outcomes of stage I lung adenocarcinoma patients. (**a**) Kaplan–Meier curves of lung adenocarcinoma patients stratified by tumour stage. Patients with higher stages tended to have worse prognosis (log-rank test *P* value <0.001 in TCGA data set, log-rank test *P*=0.0068 in TMA data set). However, the survival outcomes varied widely. (left: TCGA data set, right: TMA data set). (**b**) Kaplan–Meier curves of stage I lung adenocarcinoma patients stratified by tumour grade. Tumour grade did not significantly correlate with survival (left: TCGA data set, log-rank test *P* value=0.06; right: TMA data set, log-rank test *P* value=0.0502). (**c**) Kaplan–Meier curves of stage I lung adenocarcinoma patients stratified using quantitative image features. Image features predicted the survival outcomes. Elastic net-Cox proportional hazards model categorized patients into two prognostic groups, with a statistically significant difference in their survival outcomes in the TCGA test set (log-rank test *P* value=0.0023). (**d**) The same classification workflow was validated in the TMA data set, with comparable prediction performance. (log-rank test *P* value=0.028). (**e**) Sample image of stage I adenocarcinoma with long survival. This patient suffered from stage IB, grade 3 lung adenocarcinoma, and survived more than 99 months after diagnosis. Our classifier correctly predicted the patient as a long survivor. (**f**) Sample image of stage I adenocarcinoma with short survival. This patient suffered from stage IB, grade 3 lung adenocarcinoma, and survived less than 12 months after diagnosis. Our classifier correctly predicted the patient as a short survivor.

We next investigated the prognostic values of our quantitative feature sets. Stage I adenocarcinoma patients are known to have diverse survival outcomes (Fig. 3a). In the TCGA cohort, more than 50% of the stage I adenocarcinoma patients died within 5 years after the initial diagnosis, whereas ∼15% of the patients survived for more than 10 years. A number of studies aimed to distinguish patients with different survival outcomes with additional visual patterns^22^,^23^. However, non-systematic errors may take place using these subjective assessments, and these visual evaluations are hard to standardize^26^,^27^,^28^. It is thus difficult for human evaluators to predict survival outcomes based purely on the H&E stained microscopic slides^4^,^18^. Although higher tumour grade is thought to be associated with poorer survival outcomes^14^, this association is weak in patients with stage I lung adenocarcinoma in both TCGA and TMA data sets (log-rank test *P* value>0.05; Fig. 3b).

With an aim to provide better prognostic prediction with the H&E slides, we investigated whether our quantitative features could predict survival in stage I patients. We built elastic net-Cox proportional hazards models^46^ to select the most informative quantitative image features and calculated survival indices derived from H&E stained microscopic pathology images (see Methods). Patients were categorized into longer-term or shorter-term survivors based on their survival indices. Our model successfully distinguished shorter-term survivors from longer-term survivors in the test set (log-rank test *P* value=0.0023; Fig. 3c). Among the 60 image features selected by our methods, the top features that facilitated classification of survival outcomes included texture of the nuclei, Zernike shape decomposition of the nuclei, and Zernike shape decomposition of the cytoplasm (Supplementary Data 1).

Our approach for survival prediction was validated with images from an independent data set (the Stanford TMA database). The same image processing workflow with elastic net-Cox proportional hazards model selected a similar set of features, which also successfully distinguished longer-term survivors from shorter-term survivors in the stage I adenocarcinoma cohort (log-rank test *P* value=0.028; Fig. 3d). The patients in different survival groups did not have significantly different treatments (χ^2^-test *P* value>0.9 for neoadjuvant chemotherapy, radiation therapy and targeted molecular therapy).

Figure 3e,f show some examples of histopathology images from stage I lung adenocarcinoma patients with the same pathology grade, but with different survival outcomes. The differences in tumour cell morphology between the two histopathology images were not easily identified by visual inspection, but could be distinguished based on our quantitative image features. These quantitative features proved to be useful in predicting survival outcomes of stage I adenocarcinoma patients.

### Image features predict squamous cell carcinoma survival

**Figure 4:**
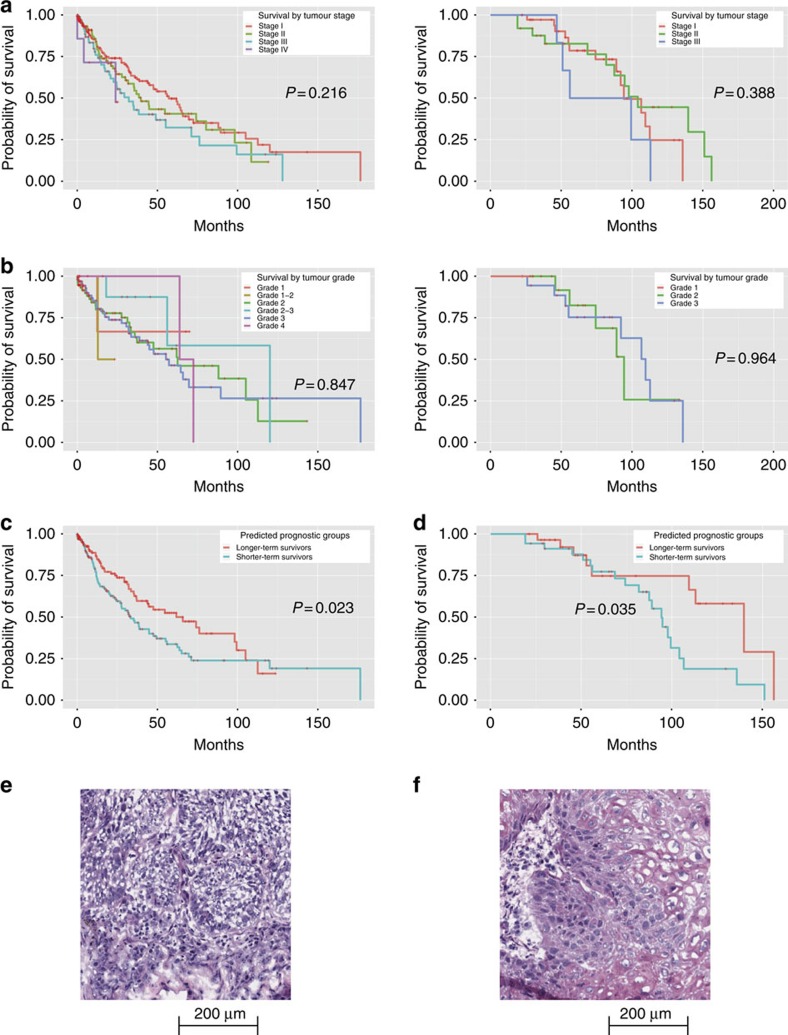
Quantitative image features predicted the survival outcomes of lung squamous cell carcinoma patients. (**a**) Kaplan–Meier curves of lung squamous cell carcinoma patients stratified by tumour stage. Although patients with higher stages generally have worse outcomes, the trend was not statistically significant (left: TCGA data set, log-rank test *P* value=0.216; right: TMA data set, log-rank test *P* value=0.388). (**b**) Kaplan–Meier curves of stage I lung squamous cell carcinoma patients stratified by tumour grade. Tumour grade did not significantly correlate with survival. (left: TCGA data set, log-rank test *P* value=0.847; right: TMA data set, log-rank test *P* value=0.964). (**c**) Kaplan–Meier curves of lung squamous cell carcinoma patients stratified using quantitative image features. The image features predicted the survival outcomes. Elastic net-Cox proportional hazards model categorized patients into two prognostic groups, with a statistically significant difference in their survival in the TCGA test set (log-rank test *P* value=0.023). (**d**) The same classification workflow was validated in the TMA data set, with comparable prediction performance. (log-rank test *P* value=0.035). (**e**) Sample image of lung squamous cell carcinoma in a patient with long survival. This patient suffered from stage I, grade 1 lung squamous cell carcinoma, and survived more than 70 months after diagnosis. Our classifier correctly predicted the patient as a long survivor. (**f**) Sample image of squamous cell carcinoma in a patient with short survival. This patient suffered from stage I, grade 1 lung squamous cell carcinoma, and only survived 12.4 months after diagnosis. Our classifier correctly predicted the patient as a short survivor.

Stage and grade alone only have limited predictive values in stratifying survival outcomes in patients with squamous cell carcinoma (log-rank test *P* value>0.2; Fig. 4a,b)^19^. To validate the generalizability of our survival prediction method to other lung cancers, we utilized similar informatics workflow incorporating image features and tumour stage to build prediction models in squamous cell carcinoma based on our quantitative image features. Our elastic net models selected 15 features and classified patients into different survival groups (log-rank test *P* value=0.023; Fig. 4c). Features most indicative of survival outcomes included Zernike shape in the tumour nuclei and cytoplasm (Supplementary Data 2).

Our prognostic methodology for squamous cell carcinoma was also confirmed in the independent Stanford TMA cohort. Elastic net-Cox proportional hazards model successfully distinguished longer-term survivors from shorter-term survivors with lung squamous cell carcinoma (log-rank test *P* value=0.035; Fig. 4d). The patients in different survival groups did not have significantly different treatments (χ^2^-test *P* value>0.71 for neoadjuvant chemotherapy, radiation therapy and targeted molecular therapy). Similarly, Zernike shape, texture and radial distribution of intensity were among the top prediction features. Figure 4e,f shows examples of histopathology images from squamous cell carcinoma patients with the same pathology stage and grade, but with different survival outcomes. As with lung adenocarcinoma, the visual features associated with survival outcomes of lung squamous carcinoma were not well established^24^,^25^, but our methodology could quantify some of the pathology patterns predictive of patient survival.

## Discussion

To our knowledge, this is the first study to predict the prognoses of lung cancer patients by quantitative histopathology features extracted from whole-slide pathology images. In this study, we designed an automated workflow that identified thousands of objective features from the images, built and evaluated machine-learning classifiers to predict the survival outcomes of lung cancer patients. We also validated our methodology using histopathology images from an independent tissue microarray database.

Previously, the vast amount of information contained in whole-slide pathology images has posed a great computational challenge to researchers. The huge dimension of the original images made it extremely difficult to manipulate, and informatics workflows requiring manual tumour tissue segmentation were not feasible for millions of image tiles. As such previous investigators have only focused on selected represented views in tissue microarrays rather than whole slides^31^,^47^. An advantage of our approach is that no additional human effort is needed in our informatics workflow other than the diagnostic labels and survival information for the training data. This makes it scalable to large amount of information contained in whole-slide pathology images. To our knowledge, this is the first study to show the utility of fully automated quantitative image features extracted from whole-slide histopathology images to predict patient survival. As such, it could provide rapid and objective survival prediction for numerous patients.

An important component of our image processing technique is the selection of the densest image tiles, as they generally contain the most cells per image. Since normal lung is composed predominantly of alveolar structures that are relatively sparse in cells, the densest image tiles typically show pathological changes, such as tumour, lymphocytic infiltration, inflammation or atelectasis—tissue regions where image feature extraction is expected to be biologically informative. We further established an automated pipeline to identify tumour-like cells and extract 9,879 features directly from the images. These features capture both the local anatomical structure (for example, shape of the cell nuclei) and more global patterns (for example, texture) of the tumour cell and tumour nuclei. As a benchmark for the utility of our objective features, machine-learning models with selected features successfully identified images with tumour cells and classified tumour types, showing that our image features could recapture the important image labels annotated by trained pathologists.

Patients with lung adenocarcinoma or squamous cell carcinoma are known to have very diverse survival outcomes. Even patients with the same stage and pathology grade can have very different survival times^18^,^19^. Indeed, patients with stage I lung adenocarcinoma exhibit a broad survival range, and clinical stage only weakly predicted the survival outcomes of lung squamous cell carcinoma patients. Historically, with the exception of pathological stage, the examination of H&E stained microscopic slides has provided limited information on patients' prognoses. Currently, morphological assessment of subtypes of well-differentiated adenocarcinoma or squamous cell carcinoma in combination with molecular testing yields some useful prognostic information^4^,^48^,^49^,^50^. In this study, we demonstrated that the extracted quantitative morphological features in the H&E stained slides, including Zernike shape features, predicts patient survival. These quantitative image features are generally difficult to spot by manual inspection, but computerized methods can efficiently and effectively identify such features. Since H&E stained images are routinely prepared and reviewed in current clinical practice, our classifiers could be efficiently applied to routine practice.

We validated our informatics framework for survival prediction by an independent TMA data set, demonstrating the generalizability of our approach. We leveraged elastic net-Cox proportional hazards models, which are computationally efficient, and are capable of reducing the number of parameters in the models effectively and handling right-censored survival data. This method is well-suited for analysing large amounts of data and large number of features in our analysis. Accurate prognostic prediction generated by our models can guide clinical decision making and enhance precision medicine.

We also investigated the top features associated with prognosis in lung adenocarcinoma and squamous cell carcinoma. In the adenocarcinoma group, the primary prognostic features that distinguished longer-term survivor from shorter-term survivors included Zernike shape features of the nuclei and cytoplasm and nuclei texture features. For each tumour cell, Zernike shape features of the nucleus were generated first by identifying the circle of the smallest diameter that covers the tumour nucleus, setting all pixels within the tumour nucleus to one and background to zero, and then decomposing the resulting binary image into Zernike polynomials, where the coefficients serve as features. Texture features quantified the correlations between nearby pixels within the regions of interest. This showed that nuanced patterns of nuclear shape are important determinants of patient prognosis. In the squamous cell carcinoma group, the most important features also included Zernike shape features of the nuclei. This showed that both local anatomical structures (for example, shape of cell nuclei and cytoplasm) and global patterns of the tumour cell nucleus (for example, texture of the nuclei) are associated with survival outcomes.

Machine-learning techniques have previously been shown to be useful in predicting patient prognosis in several cancers and pre-cancerous lesions^31^,^32^,^34^,^51^. For instance, researchers have developed computerized morphometry to distinguish different grades of epithelial dysplasia in Barrett's esophagus^34^, and other groups of investigators associated features in the stromal components with the prognosis of breast cancer^31^. In this study, we demonstrated that through incorporating multiple image databases, selecting the most informative features and optimizing classifiers, we are able to predict the prognosis for a cancer with diverse histopathology patterns. Our machine-learning models were trained and tested on images contributed by more than 20 medical centres, which reduces the systematic bias of any single image source. Our results also showed that the classification performance is not very sensitive to the choice of machine-learning models.

One limitation of this study is that cases submitted for TCGA and TMA databases might be biased in terms of having mostly images in which the morphological patterns of disease are definitive, which could be different from what pathologists encounter at their day-to-day practice. For instance, pathologists reviewed many slides and microscopic views, and only uploaded the most representative views to the TMA database. Although histopathology images with typical pathological patterns might be helpful in generating machine-learning models, how these diagnostic models performed in the actual clinical settings remain to be explored. In addition, certain semi-quantitative pattern assessments of adenocarcinoma, such as acinar or papillary, were not available in either databases. Future research could integrate quantitative image features along with a richer set of qualitative and semi-quantitative annotations. In addition, as the universal standard for digitalizing histopathology images is not yet established, retraining of prediction models is required for data sets with different levels of magnification. Another limitation is that this study only focused on H&E stained images. The clinical utility of integrating quantitative features from immunochemical stained images or molecular data remain to be established.

In summary, we demonstrate that histopathology image classifiers based on quantitative features can successfully predict survival outcomes of lung adenocarcinoma and lung squamous cell carcinoma patients. This capability is superior to the current practice utilized by pathologists who assess the images in terms of tumour grade and stage. Investigating the objective features associated with survival also provides insights for histopathology studies. Similar approaches may be applied to the pathology of other organs. Our methods could facilitate prognostic prediction based on the routinely collected H&E stained histopathology slides, thereby contributing to precision oncology and enhance quality of care.

## Methods

### Histopathology image sources

A total of 2,186 whole-slide H&E stained histopathology images were obtained from TCGA^37^,^38^, which included samples from 515 lung adenocarcinoma patients and 502 lung squamous cell carcinoma patients. All images were included for image processing and analysis. All tumour samples were gathered by surgical excision. Lymph nodes were assessed by pathology evaluation. R-status and adjuvant/neoadjuvant treatment status were determined by reviewing the clinical notes. For every image, the associated pathology report and clinical variables, such as demographic and survival information, were also acquired from the source database.

The whole-slide images with × 40 magnification were tiled into overlapping 1,000 × 1,000 pixels using bftools in the open microscopy environment^39^, which generated more than 10 million image tiles in total. To reduce computational time, only the 10 densest images of each image series were selected, as they contained more cells for further investigations. For each image tile, the image density was calculated as the percentage of non-white (all of the red, green, and blue values were below 200 in the 24-bit RGB colour space) pixels in that tile.

To ensure the extensibility of the developed methods, tissue microarray (TMA) images from Stanford Department of Pathology^40^ were acquired and processed as an external validation set. A total of 227 lung adenocarcinoma and 67 lung squamous cell carcinoma patients were included in this cohort, and one representative H&E stained histopathology image per patient was selected by pathologists. All images from TMA were included for further image processing.

Informed consent of the TCGA and TMA participants were obtained by the TCGA consortium^37^,^38^ and TMA investigators^40^, respectively. All images were publicly available for research purposes, and did not require institutional review board approval.

### Curation of pathology annotations and clinical variables

The pathology reports and clinical profiles of each lung adenocarcinoma and lung squamous cell carcinoma patient were acquired from TCGA as well as the Stanford TMA Database. Pathology grade (level of differentiation assessed by experienced pathologists: grade 1 is well-differentiated; grade 2 is moderate-differentiated; grade 3 is poorly-differentiated; and grade 4 is anaplastic tumour), stage, and pathology diagnosis for each patient were manually curated from the pathology reports. Demographic information, such as age, gender, ethnicity, survived days and survival status for the same set of patients were also obtained. All patients with missing stage were excluded from the survival analyses.

### Extraction of quantitative features from images

A segmentation and feature extraction pipeline was built using CellProfiler^42^,^43^. The pipeline first unmixed H&E stains using the ‘UnmixColors' module, then identified the tissue foreground from unstained background by a threshold calculated by the Otsu algorithm^41^. Regions of tissue folds were identified by their disproportionally heavy staining and discarded from further analysis. All types of cells in the images were segmented for diagnostic classification, whereas prognostic analysis focused on tumour cells only. Tumour nuclei and cytoplasm were segmented to facilitate extraction of features specific to these subcellular regions, as many manually defined nuclei and cytoplasmic patterns were known to have clinical implications^4^. ‘IdentifyPrimaryObjects' module with adaptive Otsu thresholds was utilized to identify the cell nuclei of the tumour cells. Cell bodies were then identified by the ‘IdentifySecondaryObject' module, and cytoplasm was defined as the regions in the cell outlines but outside of nuclei outlines. After the nucleus and cytoplasm of each cell were identified, 790 element features were designed with modules including ‘Measure Correlation', Measure Granularity', ‘Measure Image Area Occupied', ‘Measure Image Intensity', ‘Measure Image Quality', ‘Measure Object Intensity', ‘Measure Object Neighbours', ‘Measure Object Radial Distribution', ‘Measure Object Size Shape' and ‘Measure Texture'. Features of each cell were extracted and aggregated across the tile by mean, median, s.d. and deciles (10-quantiles) of the values. The quantitative features covered the size, shapes, pixel intensity distributions, textures of the objects, as well as the relation between neighbouring objects. These features were shown to be useful in characterizing the microscopic cell morphology^43^. The diagram of informatics workflow of histopathology image processing is shown in Supplementary Fig. 1. Because of the fact that the images from the TMA data set have different levels of magnification (about × 1.5 compared with the TCGA set), the same image-processing pipeline with adjusted size constraints were used for the TMA set. A comprehensive list of all 9,879 quantitative features could be found in Supplementary Data 3.

### Machine-learning methods for diagnosis classification

Naive Bayes classifiers^52^, SVM with Gaussian, linear, and polynomial kernels^53^, bagging, random forest with conditional inference trees^44^ and Breiman's random forest^45^ were used to conduct supervised machine-learning. Models were built and tested using R version 3.2, with ‘e1071' package for SVM and naive Bayes classifiers, package ‘ipred' for bagging, package ‘randomforest' for Breiman's random forest^45^, and package ‘party' for random forest with conditional inference trees^44^. The data sets were randomly partitioned into 70% training set and 30% test set. For each diagnostic classification task, information gain ratio measure (‘FSelector' package) was employed to select the most informative features from the training set and to avoid overfitting. To ensure the robustness of our results, the random partitioning process was repeated 20 times. The optimal number of features was determined by cross-validation on the training set. We built the models and selected the features using data only from the training set, in order to rigorously evaluate the performance of our finalized models with the untouched test set.

Two automated classification tasks were designed to evaluate the utility of the extracted features: (1) to classify images of malignancy from images of adjacent benign tissues; and (2) to distinguish lung adenocarcinoma from lung squamous cell carcinoma. The inputs to the classification algorithms were the quantitative features extracted from the images as described in the previous section, and the outputs were the predicted diagnoses groups. For tumour-type classification, the prediction results for image tiles of the same patient were aggregated.

### Machine-learning methods for prognosis prediction

Elastic net-Cox proportional hazards models (R package ‘glmnet') were built to calculate the survival index of each patient^46^. The models were trained and the features were selected on the training set. Regularization parameters were selected by 10-fold cross-validation on the training set. Elastic net-Cox proportional hazards model were built with the selected parameters, and survival indices for each patients were calculated to determine the threshold for survival group classification. The distribution of survival indices on the training examples was examined, and the median index in the training set was selected to divide patients into good and poor prognostic groups. The same threshold was used to classify patients in the test set into two predicted survival groups. We further performed sensitivity analysis on the number of discretized prognostic groups, and the results from three prognostic groups (divided by the first and second tertile of the survival indices in the training set) did not differ much from the two-group model (Supplementary Fig. 3).

### Evaluation

For diagnostic classification and distinguishing malignancy from adjacent dense normal tissues, a held-out test set from each database was utilized to evaluate the performance of each of the different classifiers. Receiver operator characteristics curves were generated and AUCs of each classifier were calculated using ‘ROCR'^54^ and ‘ggplot2'^55^ packages in R, and we used ANOVA to determine the performance difference among our best classifiers. To understand the weaknesses of our classifiers, images frequently misclassified by the classifiers were also reviewed.

For survival prediction, patients in the test set were classified into good or poor prognostic groups based on their survival indices as described above. Since there were only about 100 patients in most prediction tasks, leave-one-out cross-validation was utilized to assess the performance of our prediction models. Log-rank test was employed to examine the survival difference between different predicted groups. χ^2^-tests were employed to determine if there were any treatment (for example, chemotherapy, radiotherapy and targeted molecular therapy) differences in the predicted survival groups.

Both the diagnostic and prognostic prediction methods were validated by the TMA data set with the same evaluation methods.

### Data availability

The histopathology images, pathology reports, and clinical information of the TCGA data set are available in a public repository from the TCGA Data Portal (https://tcga-data.nci.nih.gov/tcga/). Those from the Stanford Tissue Microarray database are available at https://tma.im/cgi-bin/home.pl. All other data supporting the findings of this study are available within the article and its Supplementary Information Files or from the corresponding author upon reasonable request.

## Additional information

**How to cite this article:** Yu, K.-H. *et al.* Predicting non-small cell lung cancer prognosis by fully automated microscopic pathology image features. *Nat. Commun.* 7:12474 doi: 10.1038/ncomms12474 (2016).

## Supplementary Material

Supplementary Information Supplementary Figures 1-3 and Supplementary Tables 1-3

Supplementary Data 1 Features and Associated Weights in the Prognosis Prediction Model for Stage I Lung Adenocarcinoma Patients in the TCGA Dataset

Supplementary Data 2 Features and Associated Weights in the Prognosis Prediction Model for Lung Squamous Cell Carcinoma Patients in the TCGA Dataset

Supplementary Data 3 A Comprehensive List of Quantitative Features Extracted By the Fully Automated Histopathology Image Processing Pipeline

Supplementary Software 1 Stage I Lung Adenocarcinoma Patient Survival Prediction with Quantitative Image Features

Supplementary Software 2 Lung Squamous Cell Carcinoma Patient Survival Prediction with Quantitative Image Features

## Figures and Tables

**Table 1:** Patient characteristics of TCGA cohort.

TCGA data set	
Characteristics	Summary
Lung adenocarcinoma patients	*N*=515
Age	66.0±9.9 years
Gender	46.3% Male; 53.7% female
Number of tumour histopathology image series	*N*=831
Number of histopathology image series of adjacent benign tissue	*N*=243
Number of histopathology image tiles	*N*=5,739,972
	
**Grade**	
Grade 1	62 (12.0%)
Grade 1–2	11 (2.14%)
Grade 2	180 (35.0%)
Grade 2–3	39 (7.57%)
Grade 3	170 (33.0%)
Grade 4	5 (0.97%)
Grade unavailable	48 (9.3%)
	
**Stage**	
Stage I	254 (49.3%)
Stage II	119 (23.1%)
Stage III	81 (15.7%)
Stage IV	25 (4.9%)
Stage unavailable	36 (7.0%)
Lung squamous cell carcinoma patients	*N*=502
Age	66.7±12.4 years
Gender	74.1% Male; 25.9% female
Number of tumour histopathology image series	*N*=761
Number of histopathology image series of adjacent benign tissue	*N*=351
Number of histopathology image tiles	*N*=5,033,634
	
**Grade**	
Grade 1	9 (1.79%)
Grades 1–2	4 (0.80%)
Grade 2	198 (39.4%)
Grades 2–3	34 (6.77%)
Grade 3	225 (44.8%)
Grades 3–4	2 (0.40%)
Grade 4	9 (1.79%)
Grade unavailable	21 (4.2%)
	
**Stage**	
Stage I	242 (48.2%)
Stage II	156 (31.1%)
Stage III	87 (17.3%)
Stage IV	7 (1.4%)
Stage unavailable	10 (2.0%)

Abbreviation: TCGA, The Cancer Genome Atlas.

**Table 2:** Patient characteristics of the TMA cohort.

TMA data set	
Characteristics	Summary
Lung adenocarcinoma patients	*N*=227
Age	67.4±11.0 years
Gender	41.4% Male; 58.6% female
Number of tumour histopathology image series	*N*=227
Number of histopathology image tiles	*N*=227
	
**Grade**	
Grades 1	35 (15.4%)
Grades 1–2	0 (0%)
Grade 2	134 (59.0%)
Grades 2–3	0 (0%)
Grade 3	54 (23.8%)
Grade 4	0 (0%)
Grade unavailable	4 (1.8%)
	
**Stage**	
Stage I	121 (53.3%)
Stage II	64 (28.2%)
Stage III	41 (18.1%)
Stage IV	1 (0.4%)
Stage unavailable	0 (0%)
Lung squamous cell carcinoma patients	*N*=67
Age	68.7±8.4 years
Gender	62.7% Male; 37.3% female
Number of tumour histopathology image series	*N*=67
Number of histopathology image tiles	*N*=67
	
**Grade**	
Grade 1	4 (5.97%)
Grade 1–2	0 (0%)
Grade 2	33 (49.3%)
Grade 2–3	0 (0%)
Grade 3	28 (41.8%)
Grade 3–4	0 (0%)
Grade 4	1 (1.49%)
Grade unavailable	1 (1.49%)
**Stage**	
Stage I	36 (53.7%)
Stage II	25 (37.3%)
Stage III	6 (9.0%)
Stage IV	0 (0%)
Stage unavailable	0 (0%)

Abbreviation: TMA, Stanford Tissue Microarray.
