# Supplementary material for: Predicting non-small cell lung cancer prognosis by fully automated microscopic pathology image features
Source: Nat Commun. 2016 Aug 16;7:12474. doi: 10.1038/ncomms12474 (PMC4990706; doi:10.1038/ncomms12474)
Supplement: Supplementary Information — Supplementary Figures 1-3 and Supplementary Tables 1-3 [file ncomms12474-s1.pdf]

Supplementary Figure 1

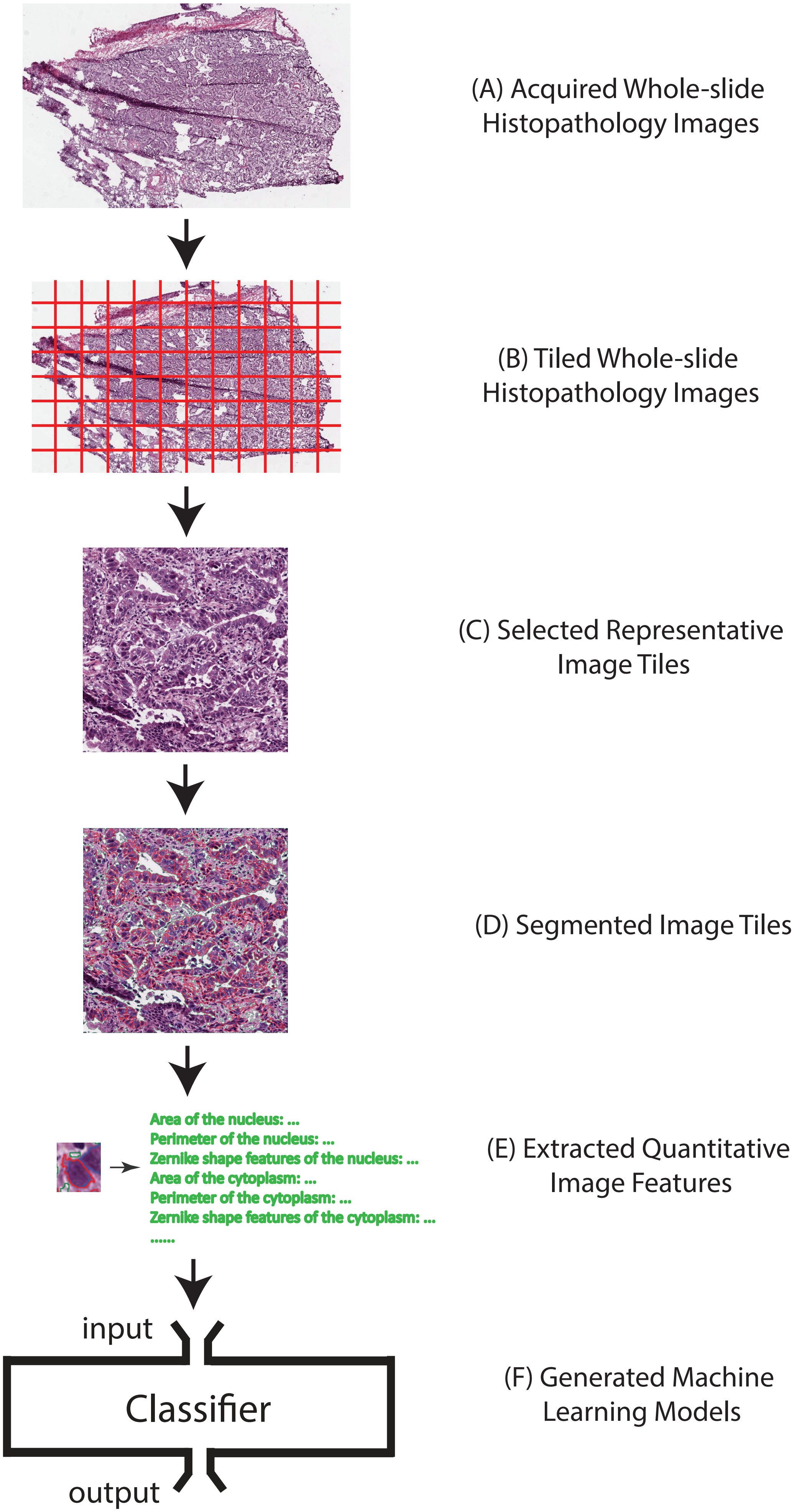

Supplementary Figure 1. (Previous page) Diagram of informatics workflow of histopathology image processing. Digital whole-slide microscopic pathology images were (A) acquired and (B) tiled with open microscopy environment tools. (C) Dense image tiles were selected, as they typically show pathological changes in the lung, such as tumor, lymphocytic infiltration, inflammation, or atelectasis. (D) Tumor nuclei and cytoplasm were segmented with modules in CellProfiler. (E) Quantitative features were extracted from the identified cell nuclei and cytoplasm, (F) and were used for classification tasks.

Supplementary Figure 2

A

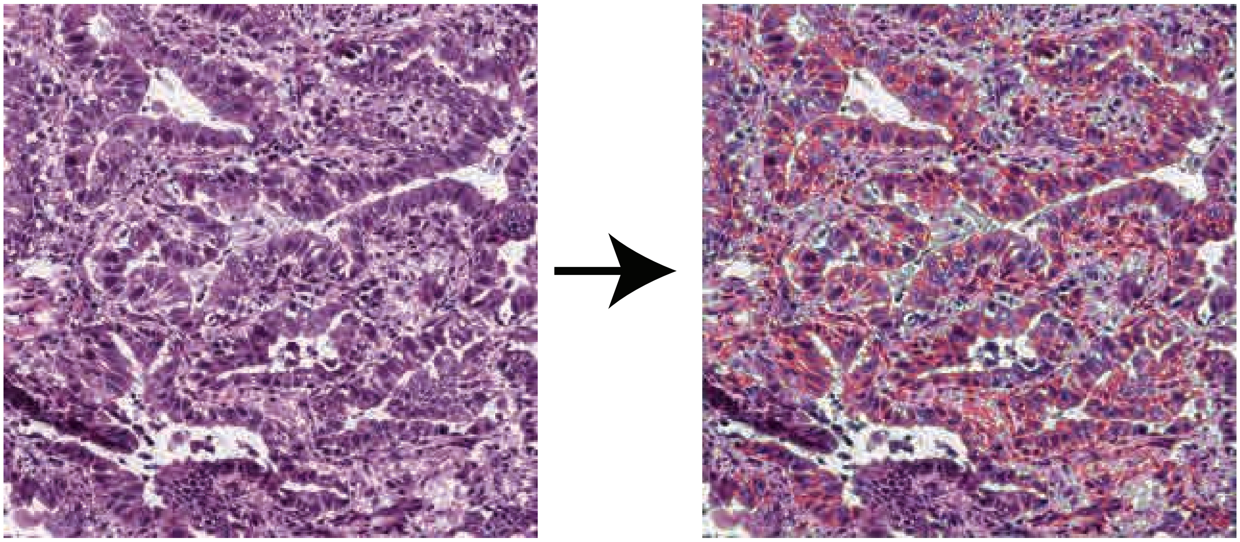

B

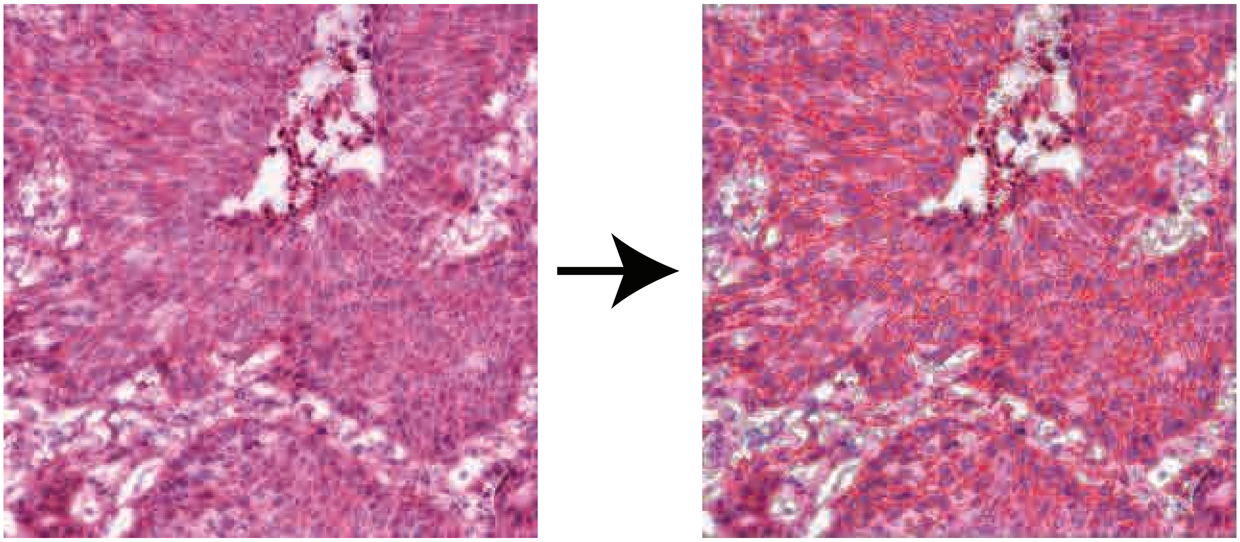

Supplementary Figure 2. (Previous page) Fully automated image segmentation algorithm identified most tumor nuclei and tumor cells from the images. (A) Raw histopathology image from a lung adenocarcinoma patient (left) and the segmented image (right). (B) Raw histopathology image from a lung squamous cell carcinoma patient (left) and the segmented image (right). In the segmented image, blue outlines indicated tumor nuclei, whereas red outlines indicated tumor cytoplasm.

Supplementary Figure 3

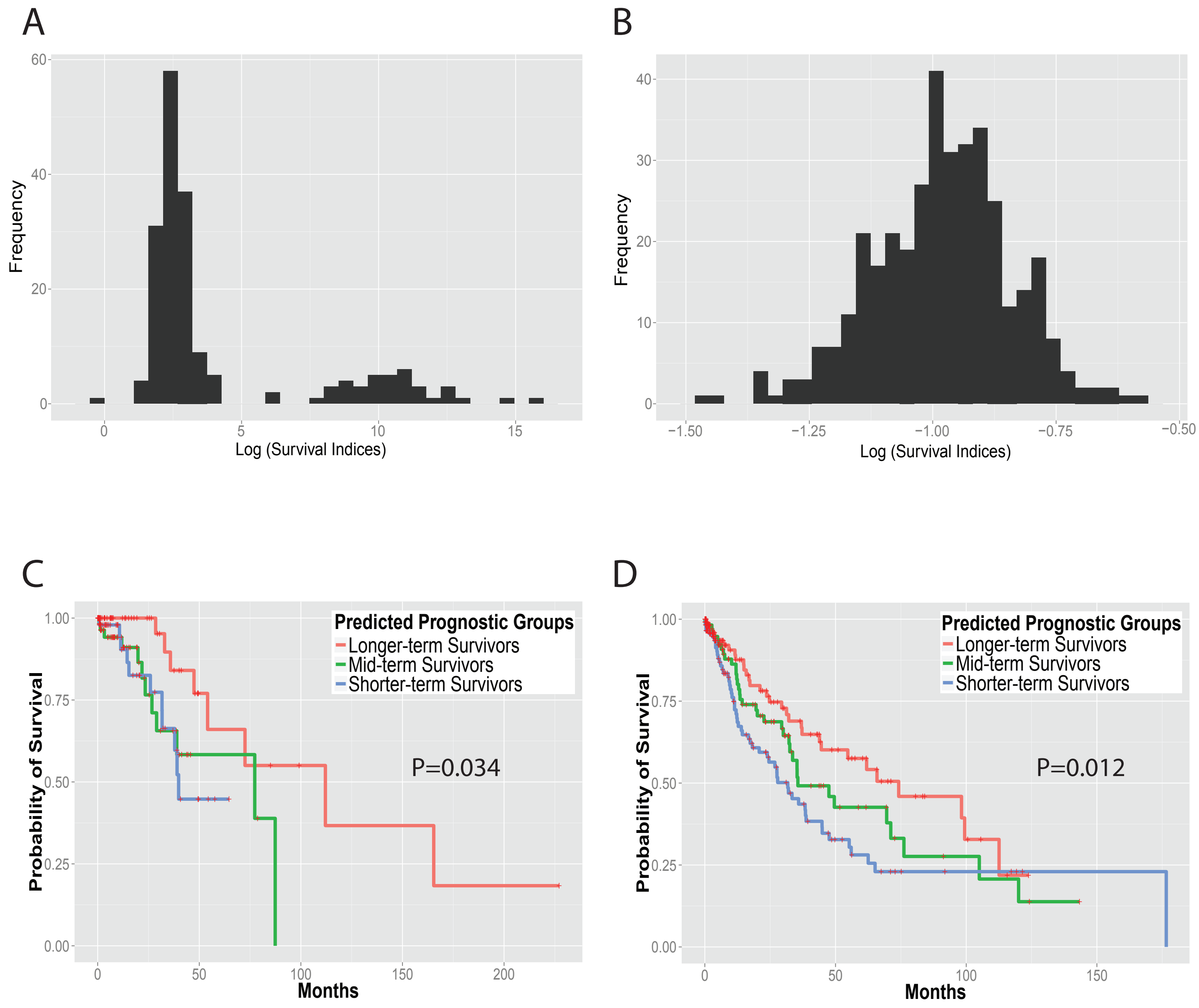

Supplementary Figure 3. The differences between the survival outcomes of different survival groups are robust for different numbers of groups. (A) Distribution of log-transformed survival indices of TCGA patients with lung adenocarcinoma. (B) Distribution of log-transformed survival indices of TCGA patients with lung squamous cell carcinoma. (C) Sensitivity analysis showed that the survival differences between groups persisted when dividing adenocarcinoma patients into three prognostic groups (P=0.034). (D) The same analysis showed that the survival differences between groups also persisted when dividing lung squamous cell carcinoma patients into three prognostic groups (P=0.012).

Supplementary Table 1. Categories of Features Included in this Study

Feature Category	CellProfiler Module	Feature Examples
Image Quality	MeasureImageQuality	Focus score, minimum intensity, maximum intensity
Image Granuarity	MeasureGranularity	Granularity of hematoxylin stained image, granularity of unmixed eosin stained image
Image Intensity	MeasureImageIntensity	Mean intensity, standard deviation of intensity, and lower and upper quartile of intensity of unmixed hematoxylin and eosin stained images
Cell Size and Shape	MeasureObjectSizeShape	Area, perimeter, Zernike shape features
Pixel Intensity of the Cell	MeasureObjectIntensity	Mean intensity, mean edge intensity, lower quartile intensity, upper quartile intensity
Radial Distribution of Pixel Intensity of the Cell	MeasureObjectRadialDistribution	Mean fractional intensity at a given radius
Cell Texture	MeasureTexture	Haralick texture features, Gabor wavelet features
Area Occupied by Cells	MeasureImageAreaOccupied	Total area occupied by the cells, total perimeter occupied by the cells
Pixel Correlation of the Cells	MeasureCorrelation	Correlation coefficients between unmixed hematoxylin and eosin stained cell regions
Neighboring Relation of the Cells	MeasureObjectNeighbors	The distance to the closest cell, the distance to the second closest cell
Nucleus Size and Shape	MeasureObjectSizeShape	Area, perimeter, Zernike shape features
Pixel Intensity of the Nuclei	MeasureObjectIntensity	Mean intensity, mean edge intensity, lower quartile intensity, upper quartile intensity
Radial Distribution of Pixel Intensity of the	MeasureObjectRadialDistribution	Mean fractional intensity at a given radius

Nuclei		
Nucleus Texture	MeasureTexture	Haralick texture features, Gabor wavelet features
Area Occupied by Nuclei	MeasureImageAreaOccupied	Total area occupied by the nuclei, total perimeter occupied by the nuclei
Pixel Correlation of the Nuclei	MeasureCorrelation	Correlation coefficients between hematoxylin and eosin stained nucleus regions
Neighboring relation of the Nuclei	MeasureObjectNeighbors	The distance to the closest nucleus, the distance to the second closest nucleus

Supplementary Table 2. Performance of Classifiers in Distinguishing Tumor Parts from Adjacent Dense Normal Tissues

Classifiers	AUCs for Distinguishing Adenocarcinoma from Adjacent Dense Normal Tissues	AUCs for Distinguishing Squamous Cell Carcinoma from Adjacent Dense Normal Tissues
SVM with Gaussian Kernel	0.85	0.88
SVM with Linear Kernel	0.82	0.86
SVM with Polynomial Kernel	0.77	0.84
Naïve Bayes Classifier	0.73	0.77
Bagging	0.83	0.87
Random Forest utilizing Conditional Inference Trees	0.85	0.87
Breiman's Random Forest	0.85	0.87

*AUC: Area Under Receiver Operating Characteristic Curves

Supplementary Table 3. Performance of Classifiers in Distinguishing Adenocarcinoma from Squamous Cell Carcinoma

Classifiers	AUCs of the TCGA Cohort	AUCs of the TMA Cohort
SVM with Gaussian Kernel	0.75	0.85
SVM with Linear Kernel	0.7	0.82
SVM with Polynomial Kernel	0.74	0.78
Naïve Bayes Classifier	0.63	0.73
Bagging	0.74	0.75
Random Forest utilizing Conditional Inference Trees	0.73	0.78
Breiman's Random Forest	0.75	0.76

*AUC: Area Under Receiver Operating Characteristic Curves
